# Supplementary material for: Vaccine-induced T cell receptor T cell therapy targeting a glioblastoma stemness antigen
Source: Nat Commun. 2025 Feb 1;16:1262. doi: 10.1038/s41467-025-56547-w (PMC11787355; doi:10.1038/s41467-025-56547-w)
Supplement: Supplementary file 1 — Supplementary Information [file 41467_2025_56547_MOESM1_ESM.pdf]

Vaccine-induced T cell receptor T cell therapy targeting a glioblastoma stemness antigen

Authors: Yu-Chan Chih[1,2,3,4], Amelie C. Dietsch[1,2], Philipp Koopmann[1,2], Xiujian Ma[2,5], Dennis A. Agardy[1,2,3,4], Binghao Zhao[1,2], Alice De Roia[1,2,3,4,6], Alexandros Kourtesakis[2,3,7,8], Michael Kilian[1,2,4,9], Christopher Krämer[1,2,4], Abigail K. Suwala[2,10,11], Miriam Stenzinger[12,13], Halvard Boenig[14,15], Agnieszka Blum[16], Victor Murcia Pienkowski[16], Kuralay Aman[1,2], Jonas P. Becker[2,17,18], Henrike Feldmann[3,4], Theresa Bunse[1,2,4], Richard Harbottle[2,6], Angelika B. Riemer[2,17,18], Hai-Kun Liu[2,5], Nima Etminan[19], Felix Sahm[2,10,11], Miriam Ratliff[19], Wolfgang Wick[2,7,8], Michael Platten[1,2,4,20,21,22], Edward W. Green[1,2], Lukas Bunse[1,2,4,22]

Affiliations:

[1]Clinical Cooperation Unit (CCU) Neuroimmunology and Brain Tumor Immunology, German Cancer Research Center (DKFZ), Heidelberg, Germany
[2]German Cancer Consortium (DKTK), DKFZ, core center Heidelberg, Germany
[3]Faculty of Biosciences, Heidelberg University, Heidelberg, Germany
[4]Department of Neurology, Medical Faculty Mannheim, Mannheim Center for Translation Neuroscience (MCTN), Heidelberg University, Mannheim, Germany
[5]Division of Molecular Neurogenetics, DKFZ, DKFZ-ZMBH alliance, Heidelberg, Germany
[6]DNA Vector Laboratory, DKFZ, Heidelberg, Germany
[7]Neurology Clinic, Heidelberg University Hospital, Heidelberg, Germany
[8]CCU Neurooncology, DKFZ, Heidelberg, Germany
[9]Ann Romney Center for Neurologic Diseases, Brigham and Women's Hospital, Harvard Medical School, Boston, MA, USA
[10]Institute for Pathology, Department of Neuropathology, Heidelberg University, Heidelberg, Germany
[11]CCU Neuropathology, DKFZ, Heidelberg, Germany
[12]Institute for Clinical Transfusion Medicine and Cell Therapy, Heidelberg, Germany
[13]Institute for Immunology, Heidelberg University Hospital, Heidelberg, Germany
[14]Faculty of Medicine, Goethe University, Frankfurt a.M., Germany
[15]Institute for Transfusion Medicine and Immunohematology, German Red Cross Blood Service Baden-Württemberg-Hessen, Frankfurt a.M., Germany
[16]Ardigen, ul. Podole 76, Kraków, Poland
[17]Division of Immunotherapy and Immunoprevention, DKFZ, Heidelberg, Germany
[18]Molecular Vaccine Design, German Center for Infection Research (DZIF), partner site Heidelberg, Germany
[19]Department of Neurosurgery, University Hospital Mannheim, Mannheim, Germany
[20]Immune Monitoring Unit, National Center for Tumor Diseases (NCT), NCT Heidelberg, a partnership between DKFZ and Heidelberg University Hospital, Heidelberg, Germany
[21]Helmholtz Institute for Translational Oncology Mainz (HI-TRON Mainz) – A Helmholtz Institute of the DKFZ, Mainz, Germany
[22]DKFZ Hector Cancer Institute at the University Medical Center Mannheim, Mannheim, Germany

Supplementary Information

1. **Supplementary Figures**
   a. **S1** *PTPRZ1* expression in TCGA and scRNA-seq datasets.
   b. **S2** Cellular states and GSC association with PTPRZ1.
   c. **S3** Validation of dominant PTPRZ1$^{1347\text{-}1355}$ TCR clonotypes.
   d. **S4** TMG design and generation of TMG-expressing cell line.
   e. **S5** PTPRZ1$^{1814\text{-}1822}$ TCR-T cells were activated and performed cytotoxicity.
   f. **S6** CD4^{+} PTPRZ1$^{1814\text{-}1822}$ TCR-T cells were also activated, and CD4^{+} and CD8^{+} could be isolated with high purity.
   g. **S7** S.c tumor model treated with i.v. ACT.
   h. **S8** I.c. tumor model treated with i.v. ACT.
   i. **S9** I.c. tumor model treated with i.v. and i.cv. ACT.
   j. **S10** PTPRZ1$^{1814\text{-}1822}$ TCR-T cells killed primary glioblastoma cells with a preference for SCCs.
   k. **S11** IPTO scRNA-seq after treatment with TCR-T cells.
2. **Supplementary Tables**
   a. **S1** Antigens of interest and control antigens encoded in the TMG.
   b. **S2** Off-targets predicted with ARDitox and their corresponding genes.
3. **Supplementary Methods**
   a. Assays
   b. Antibodies/Fluorescence labeling kits
   c. qPCR primers

Figures

Supplementary Figure 1

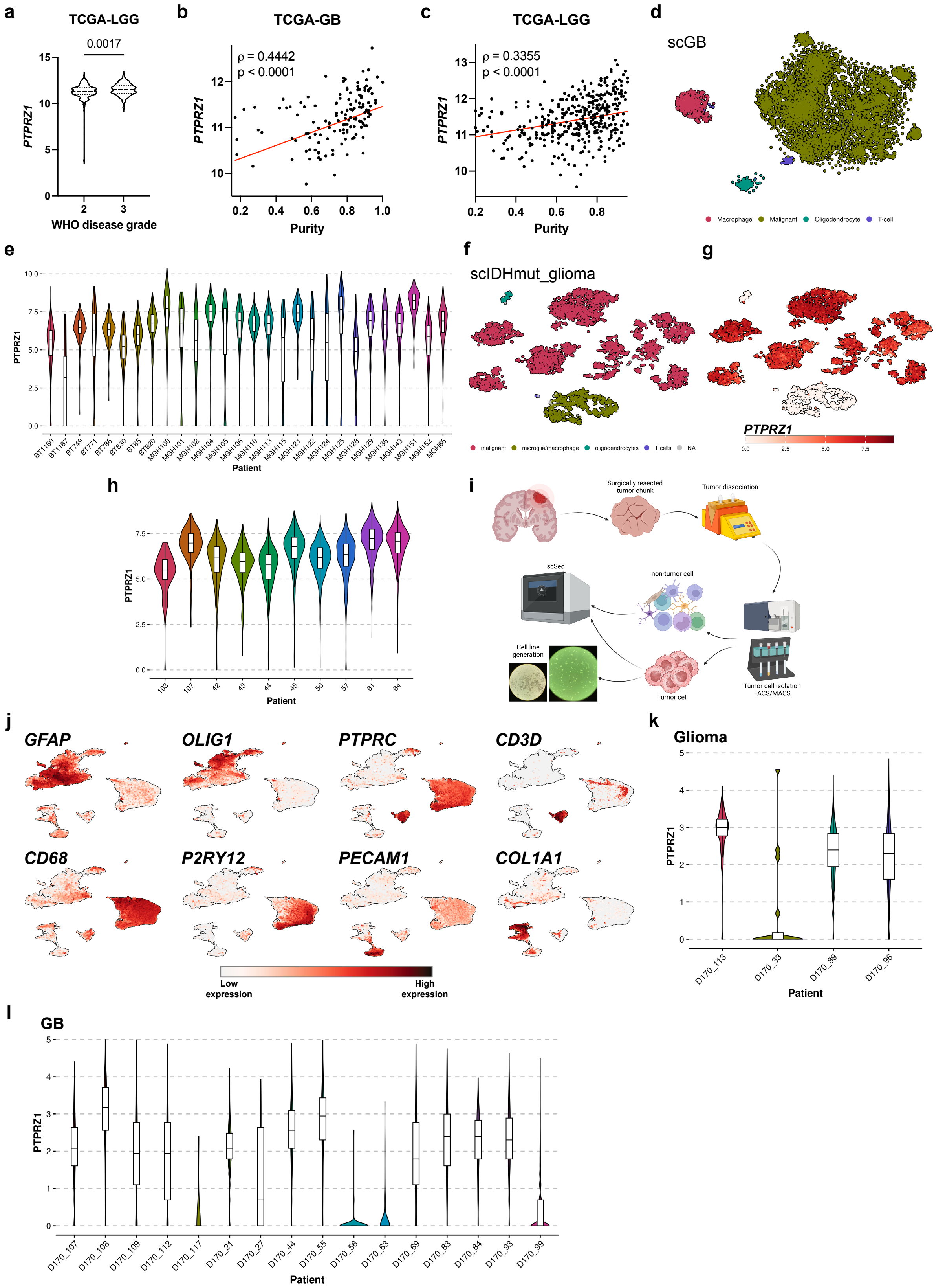

Supplementary Fig. 1. *PTPRZ1* expression in TCGA and scRNA-seq datasets. a *PTPRZ1* expression in different disease grades (grade two n=216, grade three n=241) in TCGA-LGG dataset with t-test. **b** & **c** *PTPRZ1* expression correlation with tumor purity in TCGA-GB and

LGG datasets. **d** t-SNE plot with cell type annotation for Fig. 1b. **e** *PTPRZ1* expression across glioblastoma patients from (**d**). **f** & **g** *PTPRZ1* expression in t-SNE plot with cell type annotation. **h** *PTPRZ1* expression across glioma patients. **i** Workflow from tumor tissue processing/dissociation to tumor cell isolation, cell line establishment, and single-cell sequencing. **j** Gene expression of canonical cell markers. It refers to cell types in Fig. 1c. **k** & **l** *PTPRZ1* expression across patients in both glioma and glioblastoma samples from (**i**). All the correlation tests were performed with Spearman approach. Created in BioRender. D170, P. (2025) https://BioRender.com/w70u052 (**i**).

Supplementary Figure 2

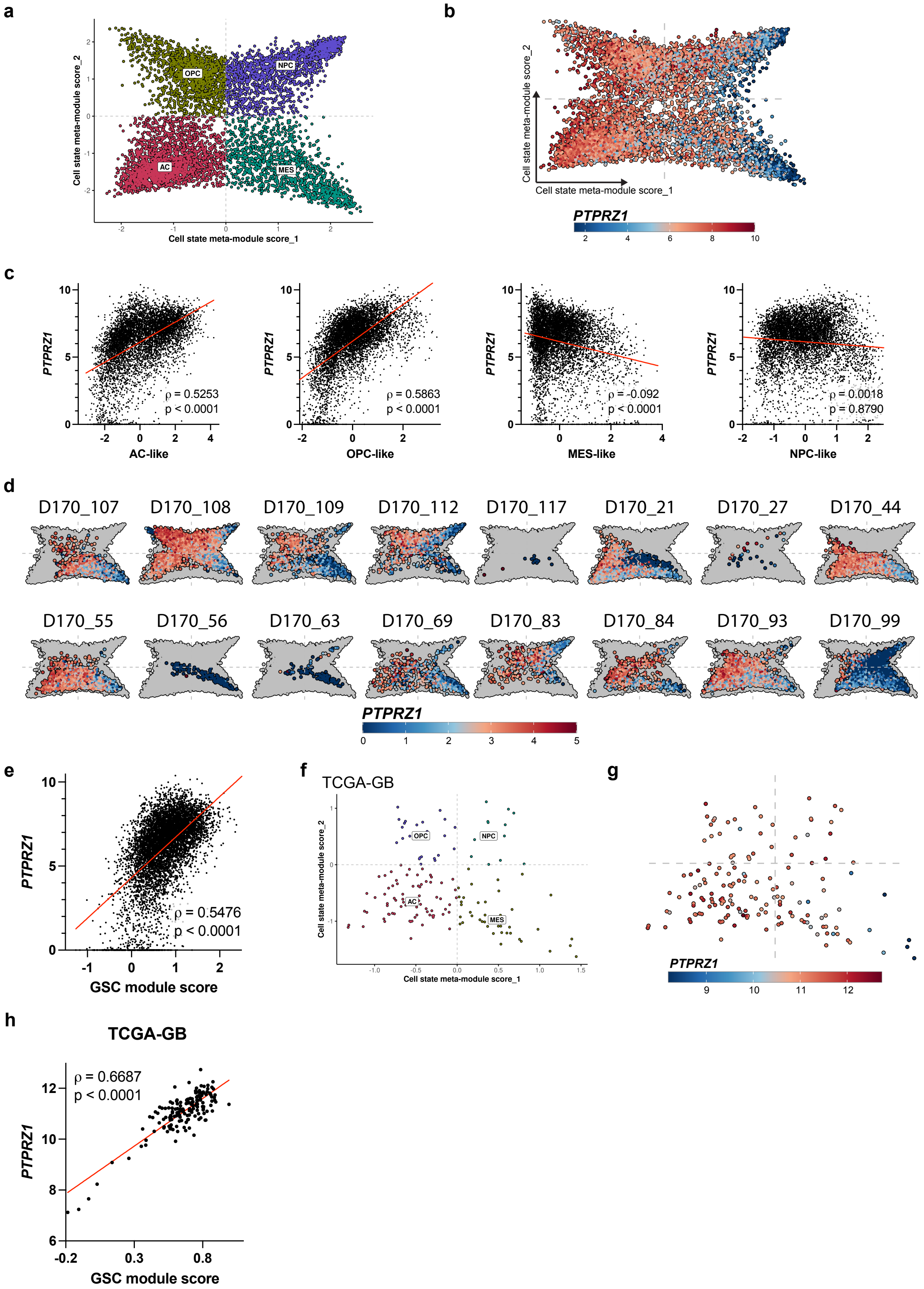

Supplementary Fig. 2. Cellular states and GSC association with PTPRZ1. a & b Distinct cellular states and *PTPRZ1* expression in different cell states in glioblastoma malignant cells in Fig. 1b. **c** Correlation of *PTPRZ1* with each cell state score. **d** Individual cancer cellular states of each glioblastoma patient from Supplementary Fig. 1i with *PTPRZ1* expression. The

overall state is in Fig. 1j, k. **e** PTPRZ1 correlation with GSC score for malignant cells in Fig. 1b and Supplementary Fig. 1d. **f** Overall cellular states of TCGA-GB tumors. **g** *PTPRZ1* expression in different cell states of TCGA-GB tumors in (**f**). **h** Correlation of GSC score and *PTPRZ1* in TCGA-GB. All the correlation tests were performed with Spearman approach.

Supplementary Figure 3

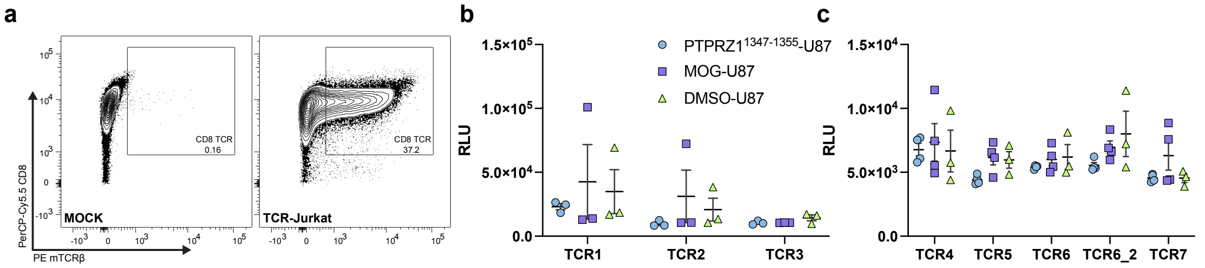

Supplementary Fig. 3. Validation of dominant PTPRZ1$^{1347\text{-}1355}$ TCR clonotypes. a Assessment of TCR transfection efficiency in Jurkat cells 48 hours post electroporation. **b** & **c** Authentication of the reactivity of dominant PTPRZ1$^{1347\text{-}1355}$ TCR clonotypes using Jurkat reporter cells from Fig. 2b. In (**b**), n(technical)=3. In (**c**), n(technical)=4 for peptide-loaded U87 and n(technical)=3 for DMSA-U87. Data are presented as mean values ± SEM.

Supplementary Figure 4

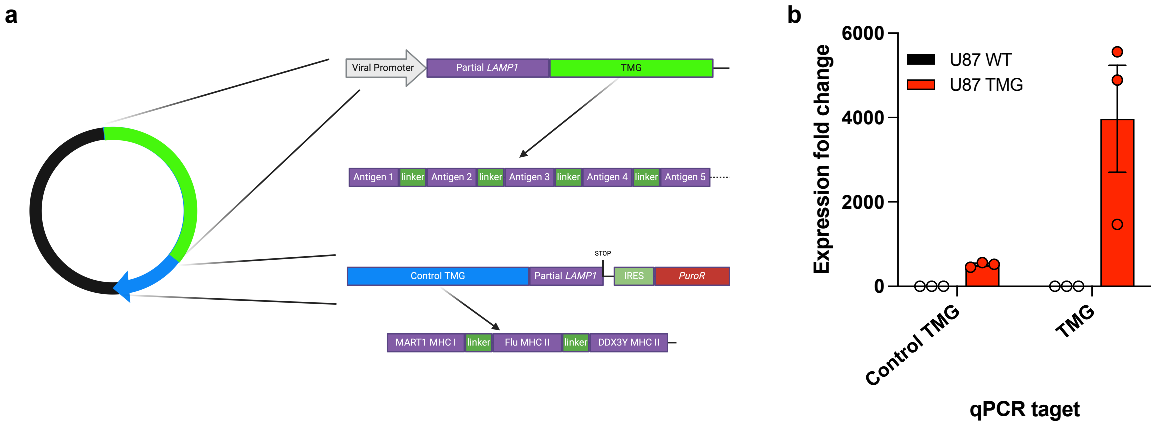

Supplementary Fig. 4. TMG design and generation of TMG-expressing cell line. a TMG design encoded in a plasmid with antigens of interest and control antigens. Antigens are listed in Supplementary Table 1. **b** Examination of TMG transcript expression in parent WT and TMG-expressing cell lines. n(technical)=3. Data are presented as mean values ± SEM. Created in BioRender. D170, P. (2025) https://BioRender.com/t89u422 (**a**).

Supplementary Figure 5

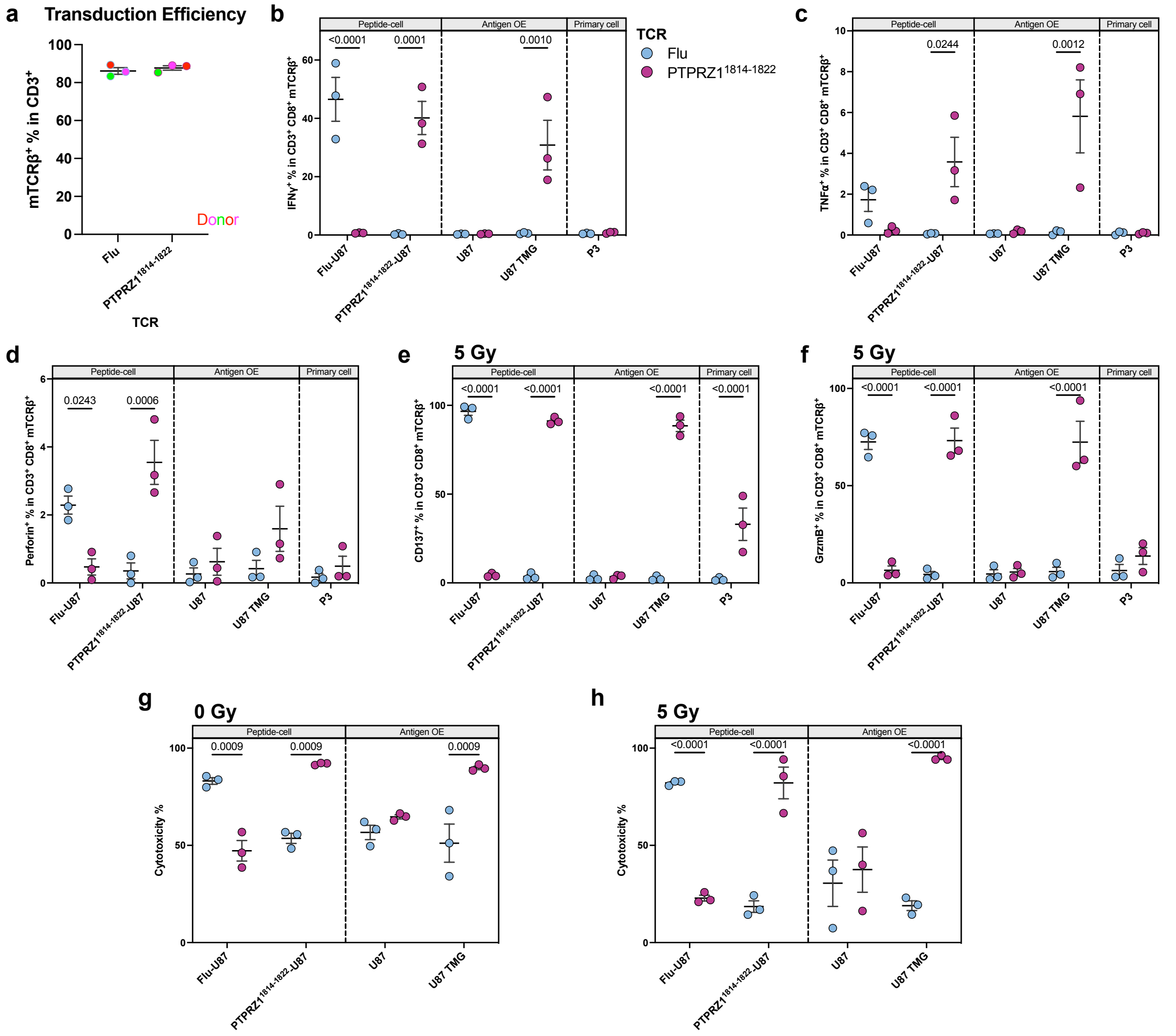

Supplementary Fig. 5. PTPRZ1$^{1814\text{-}1822}$ TCR-T cells were activated and performed cytotoxicity. a TCR expression on primary human T cells after transduction with retroviral system. **b**, **c** & **d** Cytokine- and effector protein-expressing CD8^{+} TCR-T cell percentages cocultured with various target cells. **e** & **f** Activation marker- and effector protein-expressing CD8^{+} TCR-T cell percentages cocultured with various target cells irradiated at 5 Gy. **g** & **h** Cytotoxicity of tumor cells assessed with flow cytometry cell counting with and without 5 Gy irradiation on the target cell. All were analyzed with two-way ANOVA multiple comparison corrected with Holm-Šidák method. All replicates here are biological N=3. Data are presented as mean values ± SEM.

Supplementary Figure 6

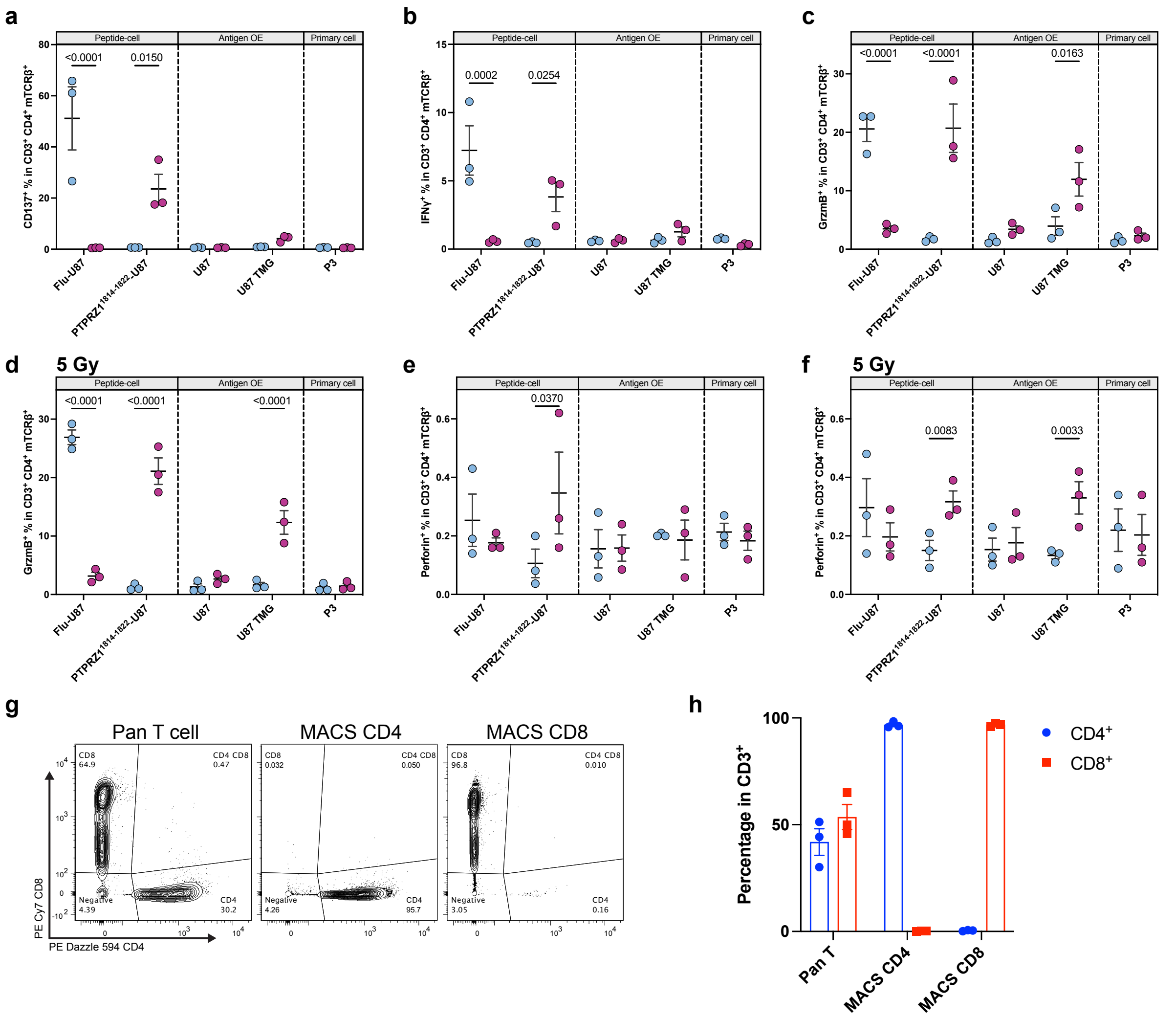

Supplementary Fig. 6. CD4⁺ PTPRZ1$^{1814\text{-}1822}$ TCR-T cells were also activated, and CD4⁺ and CD8⁺ could be isolated with high purity. a-f Activation marker-, cytokine- and effector protein-expressing CD4⁺ TCR-T cell percentages cocultured with various target cells with or without 5 Gy irradiation. All were analyzed with two-way ANOVA multiple comparison corrected with Holm-Šidák method. **g & h** Contour and summarized barplot of CD4⁺ and CD8⁺ T cells upon MACS. All replicates here are biological N=3. Data are presented as mean values ± SEM.

Supplementary Figure 7

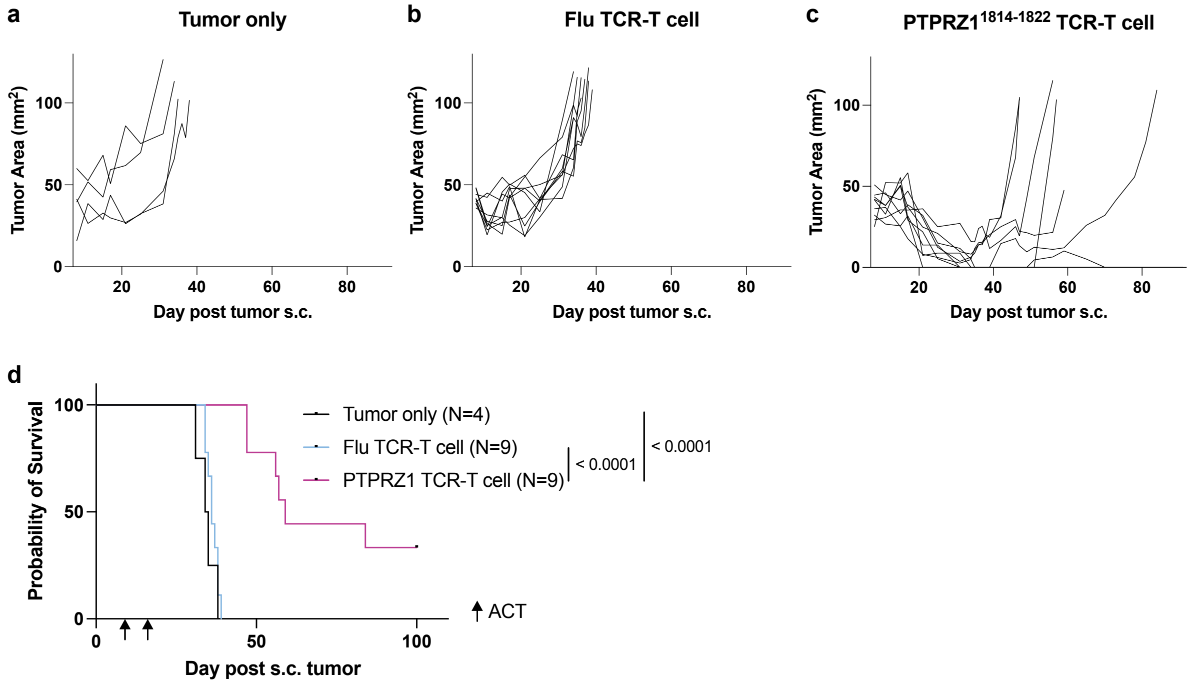

Supplementary Fig. 7. S.c tumor model treated with i.v. ACT. a-c Individual s.c. tumor growth monitoring under different treatments from Fig. 4a. **d** Overall survival of s.c. tumor-bearing mice treated with i.v. ACT, analyzed with Log-rank test.

Supplementary Figure 8

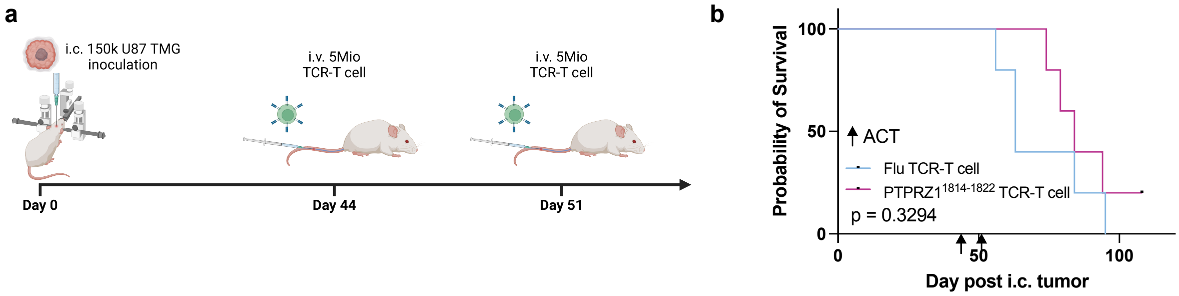

Supplementary Fig. 8. I.c. tumor model treated with i.v. ACT. a Workflow of i.v. ACT on i.c. tumor model. **b** Overall survival of i.c. tumor-bearing mice treated with i.v. ACT, analyzed with Log-rank test. N(biological)=5. Created in BioRender. D170, P. (2025) https://BioRender.com/i43f264 (**a**).

Supplementary Figure 9

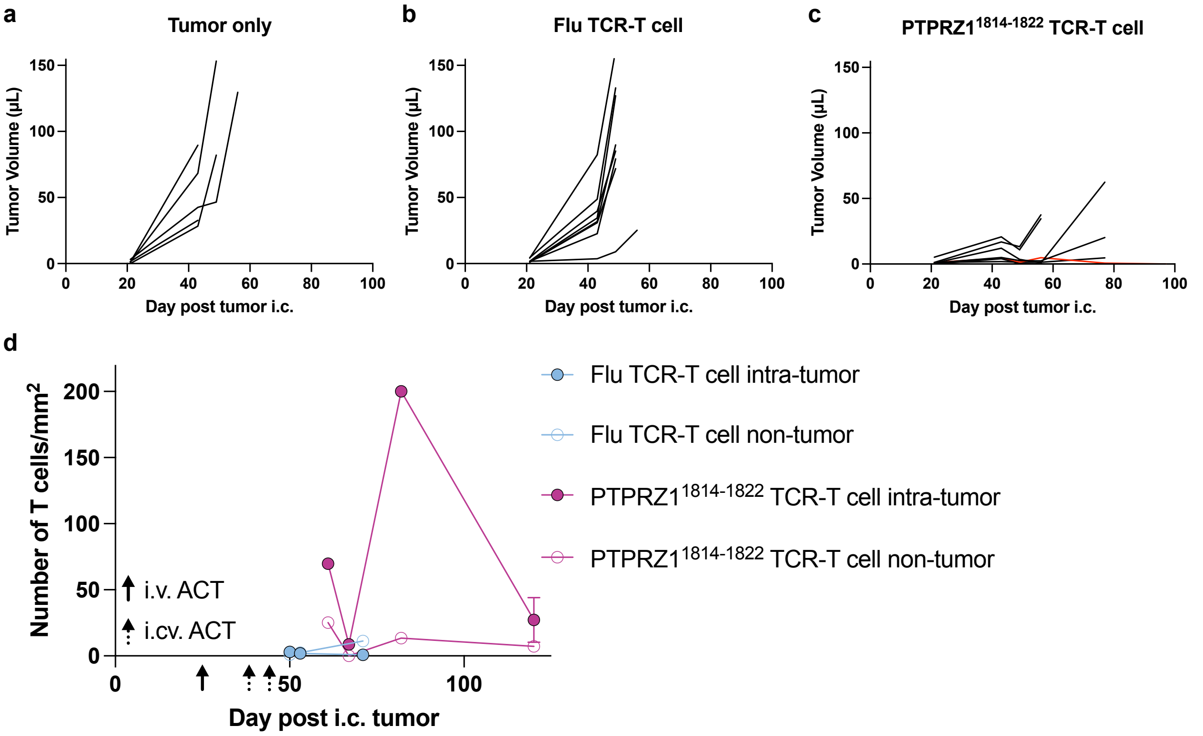

Supplementary Fig. 9. I.c. tumor model treated with i.v. and i.cv. ACT. a-c Individual i.c. tumor growth monitoring under different treatments from Fig. 4c. The survivor mouse experiencing complete regression is red-colored. **d** Transferred T cell numbers at various termination timepoints under different treatments in or outside of the tumor from Fig. 4j. Data are presented as mean values ± SEM.

Supplementary Figure 10

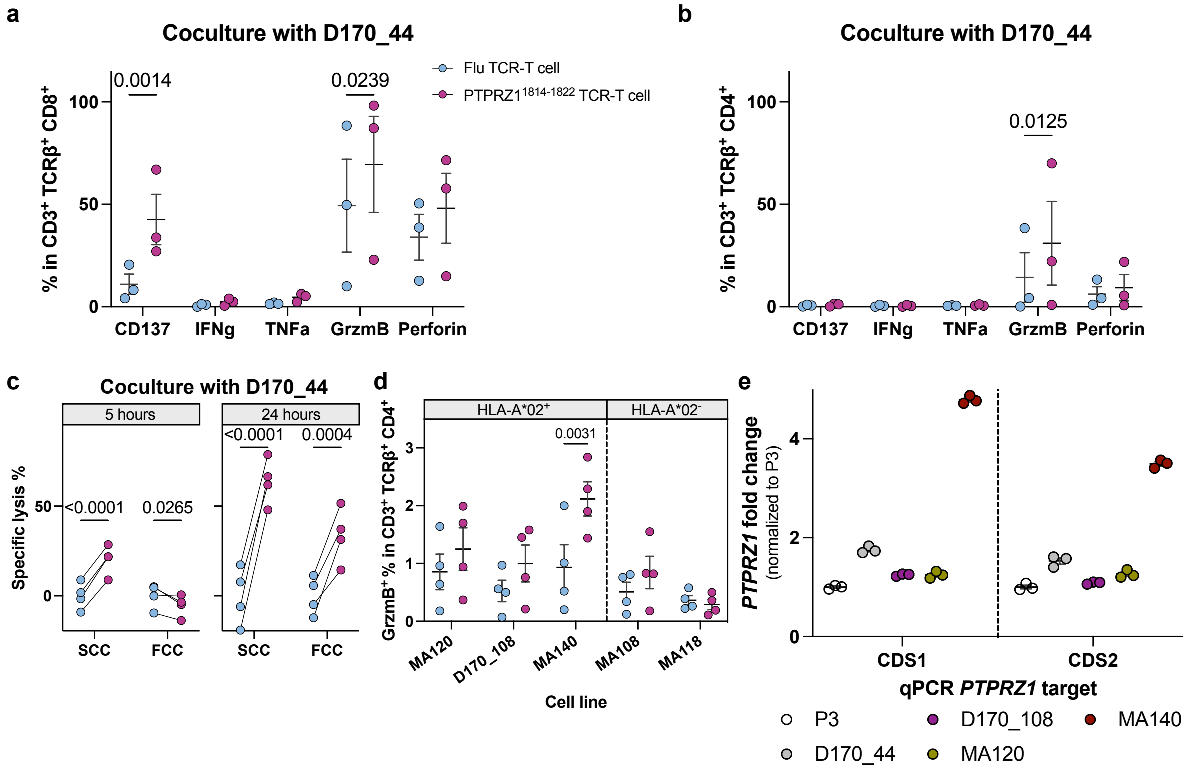

Supplementary Fig. 10. PTPRZ1$^{1814\text{-}1822}$ TCR-T cells killed primary glioblastoma cells with a preference for SCCs. a Activation markers of CD8^{+} TCR-T cells upon 24h coculture with D170_44 primary glioblastoma cell line. **b** Activation markers of CD4^{+} TCR-T cells upon 24h coculture with D170_44 primary glioblastoma cell line. **c** Cytotoxicity of SCCs and FCCs upon short-term, 5hr, or long-term, 24hr, coculture with TCR-T cells, measured with cell counting by flow cytometry. **d** Percentage of effector protein-expressing CD4^{+} TCR-T cells after 24h coculture with glioblastoma primary cell lines. **e** *PTPRZ1* expression levels across primary glioblastoma cell lines measured with RT-qPCR. The expression levels were normalized to P3 cell line, and two qPCR targets were assessed for two different CDSs of *PTPRZ1*. All were analyzed with two-way ANOVA multiple comparison corrected with Holm-Šidák method. In (**a**) and (**b**), N(biological)=3. In (**c**) and (**d**), N(biological)=4. In (**e**), n(technical)=3. Data are presented as mean values ± SEM.

Supplementary Figure 11

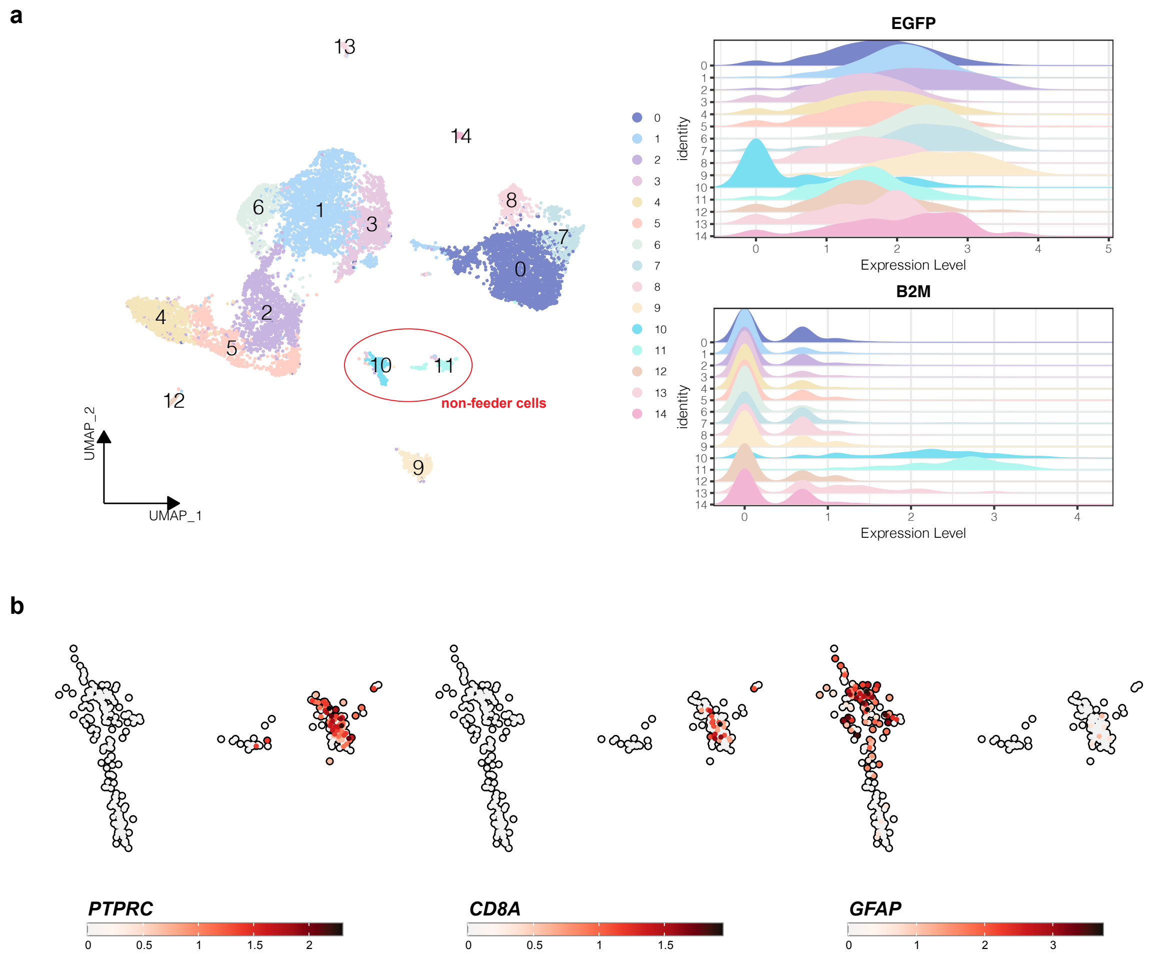

Supplementary Fig. 11. IPTO scRNA-seq after treatment with TCR-T cells. a UMAP of scRNA-sequenced IPTOs with expression of *EGFP* and *B2M* on the right. The identified non-feeder cells are shown in Fig. 6b. **b** Canonical markers of cells shown in Fig. 6b.

Tables

**TMG**	aa sequence	Synonym in GAPVAC
PTPRZ1$^{1814-1822}$	MIWEHNVEV	PTP-013
BCA	ALWAWPSEL	BCA-002
FAB7	TFGDVVAV	-
NRCAM	GLWHHQTEV	NRCAM-001
IGF2BP3	KIQEILTQV	-
PTPRZ1	AIIDGVESV	PTP-003
PTPRZ1	KVFAGIPTV	PTP-005
TNC	AMTQLLAGV	-
CSP	TMLARLASA	-
CHI	SLWAGVVVL	-
NLGN4X	NLDTLMTYV	NLGN4X-001
**Control TMG**		
MART1 MHC I	ELAGIGILTV	-
Flu MHC II	PKYVKQNTLKLAT	-
DDX3Y MHC II	CPPHIENFSDIDMGEIIMGN	-

Supplementary Table 1. Antigens of interest and control antigens encoded in the TMG.

aa sequence	Corresponding gene	Safety score	Presentation score
**MIWEHNVEV**	PTPRZ1	0	0.95
SIWRHQVEV	ZBTB21	3.24	0.71
MIWDHNAQI	PTPRG	3.26	0.51
MIWDHNAQL	PTPRZ1	3.26	0.78
MLWEHNSTI	PTPRF/D	3.33	0.83
LIYERGVEV	SLC45A2	3.33	0.69
LIMESNVEL	CEP44	4.05	0.72
EIYEKTVEV	CYFIP1/2	4.15	0.66
MVWEQGVNV	PTPN14	4.65	0.64
MIWEQKSTV	PTPN13	4.75	0.54
MIWEQKATV	PTPRC	4.75	0.72
MLNEHDFEV	BRCA1	4.88	0.95
KIYEGQVEV	RPL5	4.9	0.92

Supplementary Table 2. Off-targets predicted with ARDitox and their corresponding genes.
Cognate antigen is in bold.

Methods

a. Assays

RNA isolation

Cells were harvested and pelleted, and their RNA was isolated with PicoPure RNA Isolation kit (KIT0204, Applied Biosystems) following manufacture instructions. The eluted RNA was used directly for reverse transcription or stored at -80°C.

Reverse transcription

cDNA of the isolated RNA was generated with SuperScript II Reverse Transcriptase (18064014, Invitrogen) following manufacturer manual. The generated cDNA was 1:100 diluted in Nuclease-free water and stored at -20°C.

qPCR

2x qPCR SYBR Green master mix (SL-9902R, Steinbrenner) was used for cDNA quantification. 5 µL of diluted cDNA was mixed with 1 µl of 1 µl of 10 µM forward and 1 µl of 10 µM reverse primers and 10 µl of 2x qPCR SYBR Green master mix with sterile water to make up total 20 µl volume. The mixture was first denatured at 95°C for 1 minute, followed by 40 cycles of: 95°C for 5 seconds, 56°C for 5 seconds and 72°C for 10 seconds with melting curve on Quantstudio 3. Gene expression was calculated with ΔΔCT method with GAPDH gene as internal control.

Flow cytometry

Lineage, surface activation and subset markers were stained with the antibodies listed below in PBS. eFluor 780 fixable viability dye (65-0865-14, Invitrogen) was used following the instructions to exclude dead cells. For intracellular staining of cytokines and effector proteins, cells were incubated with 1:1000 diluted GolgiPlug and GolgiStop (555029 and 554724, BD Biosciences) for 5h. After extracellular and viability staining, cells were fixated and permeabilized with Fixation/Permeabilization solution (554714, BD Biosciences) for 30 minutes on ice, followed by 2 washes with 1x BD Perm/Wash buffer. Intracellular staining antibodies were diluted in 1x BD Perm/Wash buffer and incubated with cells for 30 minutes on ice. After another 2 washes with 1x BD Perm/Wash buffer, cells were ready for acquisition. For cell counting, 123count eBeads (01-1234-42, Invitrogen) were diluted and loaded into the well just before acquisition. Cell counts were then calculated based on the bead dilution and volume used.

LDH release

To detect LDH from killed cells, CytoTox 96 Non-Radioactive Cytotoxicity Assay kit (G1780, Promega) was used following the manufacture protocol. Briefly, 150 x 10^3 TCR-T cells and 75 x 10^3 tumor cells were cocultured for 24h unless stated otherwise. 100 µL supernatant was collected after centrifugation at 500g for 5 minutes. 50 µl of the collected supernatant was then mixed with 50 µl of CytoTox 96 Reagent and incubated at RT for 30 minutes in dark. 50 µl of Stop Solution was pipetted into the wells after incubation, and the absorbance at 490 nm was then recorded. Background of the medium and spontaneous cell leaking were subtracted.

Immunofluorescence

Human FFPE glioblastoma samples were collected at the Pathology Department in Heidelberg University hospital. Sectioned samples on slides were prewarmed at 60°C for 1h, followed by immediate immersion in histoclear/Xylol for 10 minutes and another 10-minute immersion in

fresh histoclear/Xylol. The slides then underwent 2 sequential submersions in 100% EtOH for 5 minutes each time, followed by serial hydration with 96%, 70% and 50% EtOH and lastly VE-water for 3 minutes each. Heat-mediated antigen retrieval was then done with cell conditioning solution (CC1, 950-124, Ventana) for 30 minutes in a steamer. After the slides were cooled down, they were quickly rinsed twice with PBS and permeabilized with 0.1% Tween 20 in PBS (T-PBS) for 10 minutes at RT. Blocking was done with 1:10 diluted donkey serum in T-PBS for 1h at RT. After a quick wash with T-PBS, slides were stained with primary antibodies diluted in T-PBS o/n at 4°C. On the next day, slides were washed 3 times with T-PBS for 5 minutes each time. Secondary antibodies with fluorophores were 1:200 diluted in T-PBS at incubated with the slides for 1h at RT. Subsequently, slides were washed 3 times with T-PBS for 5 minutes each time, and quickly rinsed with PBS. To quench the autofluorescence, 0.1% Sudan Black B (199664, Sigma-Aldrich) in 70% EtOH was applied for 10 minutes at RT. Lastly, the slides were washed 3 times with 0.02% Tween20 in PBS for 5 minutes each time before mounted (00-4959-52, Invitrogen).
Murine brains were harvested and embedded in OCT Compound (Sakura) before frozen at -80°C. Later, they were sectioned at 7 μm thickness, placed on slides and stored at -80°C. Before staining, slides were thaw at RT for at least 15 minutes, followed by 20-minute fixation with pre-chilled -20°C methanol. After removed from methanol, they were air-dried at RT for 5 minutes. Hydrophobic pen was used to circle sample sections on slides. Slides were then quickly rinsed with PBS and incubated with T-PBS for 30 minutes. After another PBS quick wash, blocking was done with 1:10 diluted donkey serum in T-PBS for 1h at RT. Upon a quick wash with T-PBS, slides were stained with primary antibodies diluted in T-PBS o/n at 4°C. On the next day, slides were washed 3 times with T-PBS for 5 minutes each time. Secondary antibodies with fluorophores were 1:200 diluted in T-PBS at incubated with the slides for 1h at RT. Lastly, the slides were washed 3 times with T-PBS for 5 minutes each time before mounted. The stained slides were kept at 4°C for up to a week and imaged with VS200 Research Slide Scanner (Olympus Lifescience).

b. Antibodies/Fluorescence labeling kits

Target species	Target	Fluorophore	Provider	Catalog
Human	GFAP	-	Dako	GA524
Human/Mouse	PTPRZ1	-	BD Biosciences	610179
Human	CD3	-	Dako	A0452
Human	HLA-ABC	-	BioLegend	311402
-	Fixable viability dye	eFluor 780	Invitrogen	65-0865-14
-	CellTrace Proliferation kit	Far Red	Invitrogen	C34572
Human	CD3	BV510	BioLegend	300448
Human	CD45	PerCP	BioLegend	368506
Human	CD45	Spark UV387	BioLegend	304086
Human	CD8	PerCP Cyanine5.5	BioLegend	344710
Human	CD8	PE Cy7	BioLegend	344712
Human	CD4	PE Dazzle 594	BioLegend	300548
Human	CD62L	BV711	BioLegend	304860
Human	CD45RA	BV785	BioLegend	304140
Human	CD137	PE	BioLegend	309804

Human	IFNɣ	BV421	BioLegend	502532
Human	TNFα	BV605	BioLegend	502936
Human	Granzyme B	PerCP Cyanine5.5	BioLegend	372212
Human	Perforin	Alexa Fluor 700	BioLegend	353324
Human	HLA-A2	PE	BioLegend	343306
Human	HLA-DR	BV711	BioLegend	307644
Mouse	TCRβ	PE	BioLegend	109207
Mouse	TCRβ	APC	BioLegend	109212
Mouse	CD45	Alexa Fluor 700	BioLegend	103128
Mouse	IgG	Alexa Fluor 647	Invitrogen	A31571
Rabbit	IgG	Alexa Fluor 488	Invitrogen	A21206

c. qPCR primers

Target Species	Target	Provider	Sequence
Human	GAPDH	Sigma	Fwd: TCTCTGCTCCTCCTGTTCGAC<br>Rev: TGAGCGATGTGGCTCGGCT
Synthetic	TMG	Sigma	Fwd: ACCTCCAGCTGGAGTCCATG<br>Rev: TGGTGGTGGACCTGTGTAAGAAT
Synthetic	Control TMG	Sigma	Fwd: GCCTACACCACCGCCGA<br>Rev: GCGATAGGGATCAGCATGCTG
