# Supplementary material for: Vaccine-induced T cell receptor T cell therapy targeting a glioblastoma stemness antigen
Source: Nat Commun. 2025 Feb 1;16:1262. doi: 10.1038/s41467-025-56547-w (PMC11787355; doi:10.1038/s41467-025-56547-w)
Supplement: Supplementary file 2 — Reporting Summary [file 41467_2025_56547_MOESM2_ESM.pdf]

nature portfolio

Corresponding author(s): Lukas Bunse

Last updated by author(s): Dec 22, 2024

Reporting Summary

Nature Portfolio wishes to improve the reproducibility of the work that we publish. This form provides structure for consistency and transparency in reporting. For further information on Nature Portfolio policies, see our Editorial Policies and the Editorial Policy Checklist.

Statistics

For all statistical analyses, confirm that the following items are present in the figure legend, table legend, main text, or Methods section.

n/a	Confirmed	
☐	☒	The exact sample size (n) for each experimental group/condition, given as a discrete number and unit of measurement
☐	☒	A statement on whether measurements were taken from distinct samples or whether the same sample was measured repeatedly
☐	☒	The statistical test(s) used AND whether they are one- or two-sided<br>*Only common tests should be described solely by name; describe more complex techniques in the Methods section.*
☒	☐	A description of all covariates tested
☐	☒	A description of any assumptions or corrections, such as tests of normality and adjustment for multiple comparisons
☐	☒	A full description of the statistical parameters including central tendency (e.g. means) or other basic estimates (e.g. regression coefficient) AND variation (e.g. standard deviation) or associated estimates of uncertainty (e.g. confidence intervals)
☐	☒	For null hypothesis testing, the test statistic (e.g. F, t, r) with confidence intervals, effect sizes, degrees of freedom and P value noted<br>*Give P values as exact values whenever suitable.*
☒	☐	For Bayesian analysis, information on the choice of priors and Markov chain Monte Carlo settings
☒	☐	For hierarchical and complex designs, identification of the appropriate level for tests and full reporting of outcomes
☐	☒	Estimates of effect sizes (e.g. Cohen's d, Pearson's r), indicating how they were calculated

Our web collection on statistics for biologists contains articles on many of the points above.

Software and code

Policy information about availability of computer code

Data collection	Flow cytometric data were acquired using the built-in softwares of the instruments. Immunostaining images were measured with Fiji. RT-qPCR was conducted with QuantStudio 3. scRNA-seq was performed using the Illumina platform, and sequences were aligned GRCh38 reference genome with Cell Ranger. Murine subcutaneous tumor size was measured with caliper. Murine intracranial tumor size was measured by the small animal imaging core facility at DKFZ with a Bruker BioSpec 3Tesla. Survival of the animal was noted based on weight loss and neurological behavioral symptoms. Published scRNA-seq datasets were retrieved from 10.1126/science.aai8478 and 10.1016/j.cell.2019.06.024.
Data analysis	GraphPad Prism 9.0, FlowJo, R 4.2.0, and the packages described in the materials and methods

For manuscripts utilizing custom algorithms or software that are central to the research but not yet described in published literature, software must be made available to editors and reviewers. We strongly encourage code deposition in a community repository (e.g. GitHub). See the Nature Portfolio guidelines for submitting code & software for further information.

Data

Policy information about availability of data

All manuscripts must include a data availability statement. This statement should provide the following information, where applicable:

- Accession codes, unique identifiers, or web links for publicly available datasets
- A description of any restrictions on data availability
- For clinical datasets or third party data, please ensure that the statement adheres to our policy

The use of the primary tumor cell lines specified in this manuscript is restricted by patient informed consent and institutional review board approval to this study. Single cell RNA-seq data are retrieved from 10.1126/science.aai8478 and 10.1016/j.cell.2019.06.024.

Research involving human participants, their data, or biological material

Policy information about studies with human participants or human data. See also policy information about sex, gender (identity/presentation), and sexual orientation and race, ethnicity and racism.

Reporting on sex and gender	N/A
Reporting on race, ethnicity, or other socially relevant groupings	N/A
Population characteristics	The patients were diagnosed with primary glioma or glioblastoma.
Recruitment	The patients were recruited in University Hospital Mannheim and provided written consent. Fresh glioma and glioblastoma samples were surgically resected and subjected to downstream processing, cell line establishment, and scRNA-seq.
Ethics oversight	The Mannheim Medical Faculty Ethics Committee (2017-589N-MA, 608-22, 574-23)

Note that full information on the approval of the study protocol must also be provided in the manuscript.

Field-specific reporting

Please select the one below that is the best fit for your research. If you are not sure, read the appropriate sections before making your selection.

☒ Life sciences ☐ Behavioural & social sciences ☐ Ecological, evolutionary & environmental sciences

For a reference copy of the document with all sections, see nature.com/documents/nr-reporting-summary-flat.pdf

Life sciences study design

All studies must disclose on these points even when the disclosure is negative.

Sample size	We decided sample size based on our previous experience and based on simulation with R 4.1.0 (type I error: 5%; type II error: 20%; standard deviation: 35% proportional; difference: reduction of 40%); in in vitro work with TCR-T cells, at least 3 different donors were employed
Data exclusions	Animals without tumor detection prior to treatment were excluded from the study
Replication	Different batches of donor cells provided similar and reproducible results. Animal experiments were conducted with sufficient number of mice in each group for the reproducibility of the treatment efficacy.
Randomization	Animals were randomly allocated to groups.
Blinding	Tumor size measurement was performed by core facility staff who had no knowledge of the treatment. Animal caretakers observed behavioral abnormalities without knowledge of the treatment.

Reporting for specific materials, systems and methods

We require information from authors about some types of materials, experimental systems and methods used in many studies. Here, indicate whether each material, system or method listed is relevant to your study. If you are not sure if a list item applies to your research, read the appropriate section before selecting a response.

Materials & experimental systems

n/a	Involved in the study
☐	☒ Antibodies
☐	☒ Eukaryotic cell lines
☒	☐ Palaeontology and archaeology
☐	☒ Animals and other organisms
☒	☐ Clinical data
☒	☐ Dual use research of concern
☒	☐ Plants

Methods

n/a	Involved in the study
☒	☐ ChIP-seq
☐	☒ Flow cytometry
☐	☒ MRI-based neuroimaging

Antibodies

Antibodies used	anti-hGFAP (#GA524, Dako), anti-hPTPRZ1 (#610179, BD Biosciences); anti-hCD3 (#A0452, Dako); anti-HLA-ABC (#311402, BioLegend); BV510 anti-hCD3 (#300448, BioLegend); PerCP anti-hCD45 (#368506, BioLegend); Spark UV387 anti-hCD45 (#304086, BioLegend); PerCP Cyanine5.5 anti-hCD8 (#344710, BioLegend); PE Cy7 anti-hCD8 (#344712, BioLegend); PE Dazzle 594 anti-hCD4 (#300548, BioLegend); BV711 anti-hCD62L (#304860, BioLegend); BV785 anti-hCD45RA (#304140, BioLegend); PE anti-hCD137 (#309804, BioLegend); BV421 anti-hIFNg (#502532, BioLegend); BV605 anti-hTNFa (#502936, BioLegend); PerCP Cyanine5.5 anti-hGranzymeB (#372212, BioLegend); Alexa Fluor 700 anti-hPerforin (#353324, BioLegend); PE anti-HLA-A2 (#343306, BioLegend); BV711 anti-HLA-DR (#307644, BioLegend); PE anti-mTCRb (#109207, BioLegend); APC anti-mTCRb (#109212, BioLegend); Alexa Fluor 700 anti-mCD45 (#103128, BioLegend); Alexa Fluor 647 anti-mIgG (#A31571, Invitrogen); Alexa Fluor 488 anti-rabbit IgG (#A21206, Invitrogen)
Validation	All antibodies were used according to the manufacturer instructions and quality-tested by the manufacturer.

Eukaryotic cell lines

Policy information about cell lines and Sex and Gender in Research

Cell line source(s)	U87 cell was purchased from ATCC; primary glioblastoma cell lines were from Ratliff et al., Int J Mol Sci 2022, or freshly established as per described in materials and methods
Authentication	Cell lines were purchased from ATCC, previously established or established in this study
Mycoplasma contamination	The cell lines were routinely tested and remained free of mycoplasma and other pathogens
Commonly misidentified lines (See ICLAC register)	No commonly misidentified cell lines were used in the study

Animals and other research organisms

Policy information about studies involving animals; ARRIVE guidelines recommended for reporting animal research, and Sex and Gender in Research

Laboratory animals	NXG mice were purchased from Janviers Lab, and NSG MHC KO mice were bred in-house at DKFZ, described in materials and methods
Wild animals	N/A
Reporting on sex	Both male and female mice were used due to breeding and availability. No sex-dependent results were observed.
Field-collected samples	N/A
Ethics oversight	Regional Administrative Authority Karlshruhe, Germany, file number: G-263/18 and G-130/23

Note that full information on the approval of the study protocol must also be provided in the manuscript.

Plants

Seed stocks	N/A
Novel plant genotypes	N/A
Authentication	N/A

Flow Cytometry

Plots

Confirm that:

☒ The axis labels state the marker and fluorochrome used (e.g. CD4-FITC).

☒ The axis scales are clearly visible. Include numbers along axes only for bottom left plot of group (a 'group' is an analysis of identical markers).

☒ All plots are contour plots with outliers or pseudocolor plots.

☒ A numerical value for number of cells or percentage (with statistics) is provided.

Methodology

Sample preparation	Jurkat reporter cells were kindly gifted by Prof. Steinberger; TCR-transgenic T cells were manufactured with retroviral transduction system; primary glioblastoma cells were previously established or generated here following the described protocol
Instrument	BD FACSCanto, BD LSRFortessa, Bio-Rad ZE5 Cell Analyzer
Software	FlowJo
Cell population abundance	The cell population frequencies were shown in plot
Gating strategy	Cells and counting beads were differentiated by FSC-A/SCC-A. Counting beads were further identified with their bright fluorescent signals described in the commercial manual. Single cells were gated with FSC-A/FSC-H. Viable cells were further filtered with Fixable Viability Dye eFluor™ 780. Cells were gated for their positivity of markers of interest based on the fluorescence minus one (FMO) controls. Some gatings were demonstrated in main figures.

☒ Tick this box to confirm that a figure exemplifying the gating strategy is provided in the Supplementary Information.

Magnetic resonance imaging

Experimental design

Design type	Resting state
Design specifications	N/A
Behavioral performance measures	N/A

Acquisition

Imaging type(s)	Structural
Field strength	3 Tesla
Sequence & imaging parameters	The imaging was performed with a T2 TurboRARE sequence: TE = 48 ms, TR = 3350 ms, FOV 20x20 mm, slice thickness 1 mm, averages = 3, Scan Time = 3m21s, echo spacing 12 ms, rare factor 8, slices 20, image size 192 x 192.
Area of acquisition	Whole brain

Diffusion MRI ☐ Used ☒ Not used

Preprocessing

Preprocessing software	Tumor volume was assessed by manual segmentation using Bruker Para Vision software 6.0.1.
Normalization	N/A
Normalization template	N/A
Noise and artifact removal	N/A
Volume censoring	N/A

Statistical modeling & inference

Model type and settings	N/A
Effect(s) tested	N/A

Specify type of analysis: ☐ Whole brain ☐ ROI-based ☐ Both

Statistic type for inference (See Eklund et al. 2016)	N/A
Correction	N/A

Models & analysis

n/a	Involved in the study
☒	☐ Functional and/or effective connectivity
☒	☐ Graph analysis
☒	☐ Multivariate modeling or predictive analysis
